# *Lithobates catesbeianus* (American Bullfrog) oocytes: a novel heterologous expression system for aquaporins

**DOI:** 10.1242/bio.031880

**Published:** 2018-03-12

**Authors:** Jessica Kabutomori, Olivia Beloto-Silva, R. Ryan Geyer, Raif Musa-Aziz

**Affiliations:** 1 Department of Physiology and Biophysics, Institute of Biomedical Sciences, University of Sao Paulo, Sao Paulo 05508-900, Brazil; 2 Department of Health Sciences, Paulista University (UNIP), Sao Paulo 06542-001, Brazil; 3 Department of Biochemistry, Institute of Chemistry, University of Sao Paulo, Sao Paulo 05513-970, Brazil

**Keywords:** *Lithobates catesbeianus*, American Bullfrog, Oocytes, Aquaporins, Water permeability, Xenopus laevis, Heterologous expression system

## Abstract

*Xenopus laevis* oocytes are a valuable tool for investigating the function of membrane proteins. However, regulations around the world, specifically in Brazil, render the import of *Xenopus laevis* frogs impractical, and, in some cases, impossible. Here, as an alternative, we evaluate the usefulness of the North American aquatic bullfrog *Lithobates catesebeianus*, which is commercially available in Brazil, for the heterologous expression of aquaporin (AQP) proteins. We have developed a method that combines a brief collagenase treatment and mechanical defolliculation for isolating individual oocytes from *Lithobates* ovaries. We find that they have a similar size, shape, and appearance to *Xenopus* oocytes and can tolerate and survive following injections with cRNA or water. Furthermore, surface biotinylation, western blot analysis, and measurements of osmotic water permeability (*P*_f_) show that *Lithobates* oocytes can express AQPs to the plasma membrane and significantly increase the *P*_f_ of the oocytes. In fact, the *P*_f_ values are similar to historical values gathered from *Xenopus* oocytes. Due to the presence of a mercury sensitive cysteine (Cys or C) in the throat of the water channel, the *P*_f_ of oocytes expressing human (h) AQP1, hAQP1_FLAG_ [FLAG, short protein tag (DYKDDDDK) added to the N-terminus of AQP1], hAQP8, and rat (r) AQP9 was inhibited with the mercurial compound p-chloromercuribenzene sulfonate (pCMBS), whereas AQPs lacking this Cys – hAQP1_C189S_ mutant [residue Cys 189 was replaced by a serine (Ser or S)] and hAQP7 – were mercury insensitive. Contrary to previous studies with *Xenopus* oocytes, rAQP3 was also found to be insensitive to mercury, which is consistent with the mercury-sensitive Cys (Cys 11) being located intracellularly. Thus, we consider *Lithobates* oocytes to be a readily accessible system for the functional expression and study of membrane proteins for international researchers who do not currently have access to *Xenopus* oocytes.

## Abstract (precis)

**Summary:** A unique and highly available heterologous expression system using *Lithobates catesbeianus* oocytes has been developed for researchers who have restricted or prohibited access to *Xenopus laevis* frogs and/or oocytes.

## INTRODUCTION

The use of *Xenopus laevis* oocytes as a viable heterologous expression system was first demonstrated 46 years ago (Gurdon et al., 1971). Eleven years later, it was shown that membrane proteins could also be translated and inserted into the oocyte plasma membrane (Miledi et al., 1982a,b), thus conferring functional activities that are otherwise absent in water-injected control oocytes. Since then, numerous publications have utilized this system to study the transport of a wide variety of substances (Musa-Aziz et al., 2010). One protein family, of particular relevance to this study, which has been studied extensively using *Xenopus* oocytes, is the aquaporin family of water channel proteins (Geyer et al., 2013a; Preston et al., 1992).

The landmark study by Preston et al. using *Xenopus* oocytes (Preston et al., 1992) definitively expressed and characterized the water transport properties of CHIP28 (channel-forming integral protein of 28 kDa), which is now known as aquaporin 1 (AQP1), and also showed that this protein is mercury sensitive. By systematically mutating each cysteine (Cys or C) residue in AQP1 to a non-sulfhydryl containing amino acid serine (Ser or S), this group also discovered that Cys 189, located in the throat of the water channel aquapore, near the second asparagine-proline-alanine (NPA) motif, and accessible from the extracellular surface, is the mercury-sensitive Cys residue of AQP1 (Preston et al., 1993). Since this discovery, 12 more aquaporin (AQP) proteins have been cloned and functionally characterized, most of which are also mercury sensitive, with the exceptions of AQP4 (Jung et al., 1994) and AQP7 (Ishibashi et al., 1997b). Despite the similar primary amino acid sequences and highly conserved secondary, tertiary, and quaternary structures, each AQP possesses diverse transport properties. For example, in addition to transporting water, AQPs have been shown to transport glycerol, ions, hydrogen peroxide, dissolved gases (carbon dioxide and ammonia), and arsenic (Geyer et al., 2013a). It has been shown that, in many cases, the localization of AQPs to specific organs or cell types is largely based on the transport specificities of the AQPs. While the *Xenopus* oocyte system has been instrumental in the growth and progress of the AQP field, it is not readily available to some researchers. For example, the state of California requires researchers to obtain a license to import the *Xenopus laevis* frog into the state (California Natural Resources Agency Department of Fish and Wildlife, 1984). Additionally, the import of *Xenopus* frogs and oocytes to the South American country of Brazil is tightly regulated and extremely difficult, due to the fear of the frogs escaping and becoming an invasive species, which could wreak havoc on the tropical ecosystem. Therefore, an alternative to the *Xenopus* system, using a frog that is not tightly regulated or prohibited needs to be developed and characterized.

Previous studies have shown that oocytes from other species of frogs can be utilized for the functional characterization of membrane transporters and channels. For example, *Xenopus borealis* oocytes have been used to study neuronal ion channels (Cristofori-Armstrong et al., 2015), *Bufo marinus* – indigenous to south and mainland Central America – oocytes have been employed for evaluating various membrane transporters and ion channels (Markovich and Regeer, 1999; Vargas et al., 2004), and *Bufo arenarum* – native to Argentina – oocytes have been characterized for endogenous ion currents and usefulness in expressing urea transporters (Cavarra et al., 2003; Silberstein et al., 2003). Here, we have identified a species of frog, *Lithobates catesbeianus* – also known as the American Bullfrog – that is native to North America, but was first introduced to Brazil in the 1930s (Both et al., 2011). Due to the abundance of and accessibility to *Lithobates*, the aim of this work is to evaluate the usefulness and viability of *Lithobates* oocytes as an alternative model system for heterologous expression of AQPs.

As a proof of concept, we developed an oocyte isolation procedure, which employs a brief incubation in purified collagenase to release individual *Lithobates* oocytes, followed by mechanical removal of the innermost follicular cells that create a barrier between the oocyte and the external environment. These oocytes were injected with cRNA encoding for human (h) AQP1, hAQP1_FLAG_, hAQP1_C189S_ mutant (AQP1 with the mercurial target Cys 189 mutated to a Ser), rat (r) AQP3, hAQP7, hAQP8 or rAQP9, monitored for surface expression (Geyer et al., 2013a), assessed for osmotic water permeability (*P*_f_), and evaluated for mercury sensitivity. We conclude that *Lithobates* oocytes are a viable heterologous expression system for AQPs, and can be used by researchers that have restricted access to *Xenopus* oocytes, or be employed as an alternative expression system to researchers that study AQPs. Future studies will be geared towards the applicability of *Lithobates* oocytes with other membrane proteins and assays (e.g. voltage clamp, patch clamp, intracellular and extracellular pH measurements).

## RESULTS

### Blood parameters

Before starting to develop a method for isolating oocytes from *Lithobates catesbeianus* ovaries, it was important to measure some serum chemistry parameters to make sure that (1) the solutions typically used with *Xenopus* oocytes would be compatible with *Lithobates* oocytes, and (2) *Lithobates* oocytes would respond well to the protocols used to isolate the *Xenopus* oocytes. The serum chemistry values of *Lithobates catesbeianus* are also important basic information that needs to be available to other researchers.

The arterial blood gas analysis (pCO_2_, HCO_3_^−^ and pH), osmolarity, and ion concentrations (Na^+^, Cl^−^, K^+^) were measured in arterial blood collected from *Lithobates* frogs and compared to values reported in the literature for *X. laevis*, and a summary of the results are presented in Table 1. Due to the similarities in the values, especially with regards to the osmolarity and pH, it was concluded that the solutions typically used for *Xenopus* oocyte isolation and storage would not need to be modified. Next, we evaluated the effect of using collagenase in the oocyte isolation procedure.

**Table 1.**
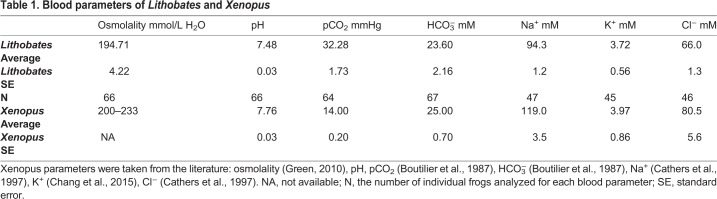
**Blood parameters of *Lithobates* and *Xenopus***

### Oocyte isolation

A typically *Xenopus* oocyte isolation process involves incubating the ovary fragments that include oocytes, connective tissue, blood vessels and follicular cells in 2 mg/ml Type IA collagenase for 40 min. This enzymatic digestion liberates individual oocytes from the ovary and from their follicular layers, leaving the vitelline membrane that surrounds the plasma membrane intact. The vitelline membrane helps to maintain the shape of the oocyte and renders the oocyte more resistant to manipulations.

However, *Lithobates* oocytes lysed and/or died when incubated with 2 mg/ml Type IA collagenase for 40 min. Attempts were made to optimize the Type IA collagenase incubation conditions by reducing the collagenase concentration (from 2 mg/ml to 1 mg/ml, 0.5 mg/ml, 0.25 mg/ml or 0.1 mg/ml) and varying the duration of the digestion process [from 40 min to 20 min, 10 min or 5 min (a brief 5 min treatment was applied to both the 0.25 mg/ml and 0.1 mg/ml concentrations of Type IA collagenase)]. Under all conditions employed, unhealthy nonviable *Lithobates* oocytes were obtained. Therefore, the efficacy and usefulness of other collagenases was investigated. Similar results were obtained with Type II collagenase (Sigma-Aldrich) and Type V collagenase (Sigma-Aldrich) at 1 mg/ml in 0-Ca^2+^ ND-96 solution.

**Fig. 1.**
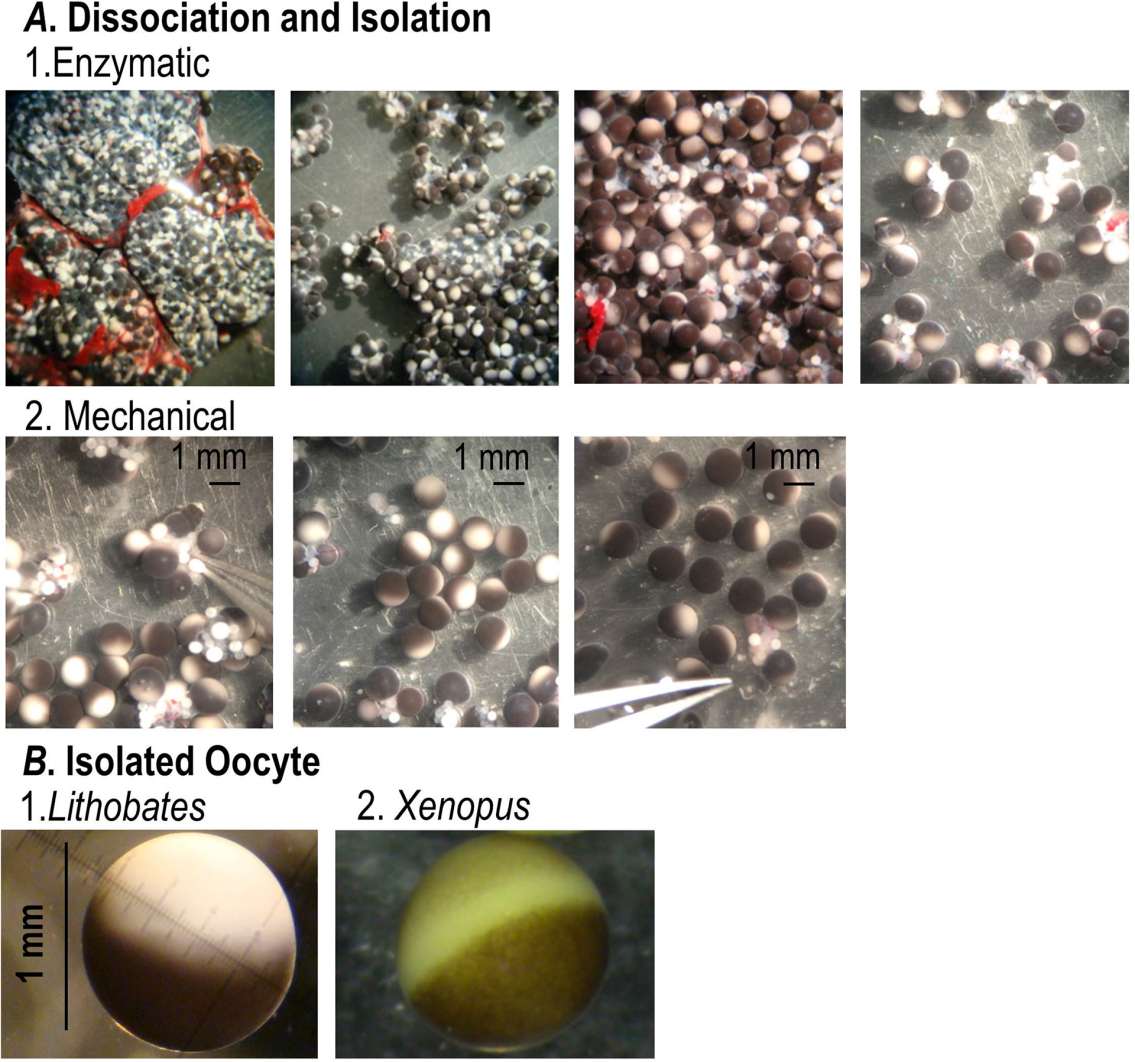
**Process of oocyte isolation.** (A) 1. Enzymatic and mechanical dissociation of *Lithobates* oocytes. First, the ovary fragments were cut into small pieces and treated with Type VII Collagenase (0.25 mg/ml) for 5 min. 2. After this first enzymatic dissociation, healthy stage V-VI oocytes were mechanically isolated with tweezers. (B) Comparison between a defolliculated isolated *Lithobates* oocyte and a defolliculated isolated *Xenopus* oocyte. Note: the image of the *Xenopus* oocyte was adapted from Batra (2016).

Ultimately, it was found that a brief 5 min treatment with the purified Type VII collagenase (0.25 mg/ml) digested the extracellular connective tissues, while leaving the innermost layer of follicular cells still attached to the exterior of the oocyte. After stopping the enzymatic digestion, the remaining follicular cells were manually removed, without disrupting the vitelline membrane. This combination of methodologies yielded healthy, fully defolliculated individual oocytes that appeared the same as they did in the ovary. Fig. 1A and B show the entire isolation process. The isolated *Lithobates* oocytes have about the same size, shape, and appearance as *Xenopus* oocytes: they are large (∼1.0 mm in diameter) with a well-defined dark animal pole (where the nucleus is located), a weakly pigmented vegetal pole (containing the majority of the yolk proteins), and a very faint, sometimes unnoticeable, unpigmented equatorial belt, between the two poles. These *Lithobates* oocytes persisted in OR3 media for 5 days without signs of cell lysis, discoloration or infection. Having established a feasible isolation procedure, it was then necessary to evaluate the usefulness of *Lithobates* oocytes as a heterologous expression system.

**Fig. 2.**
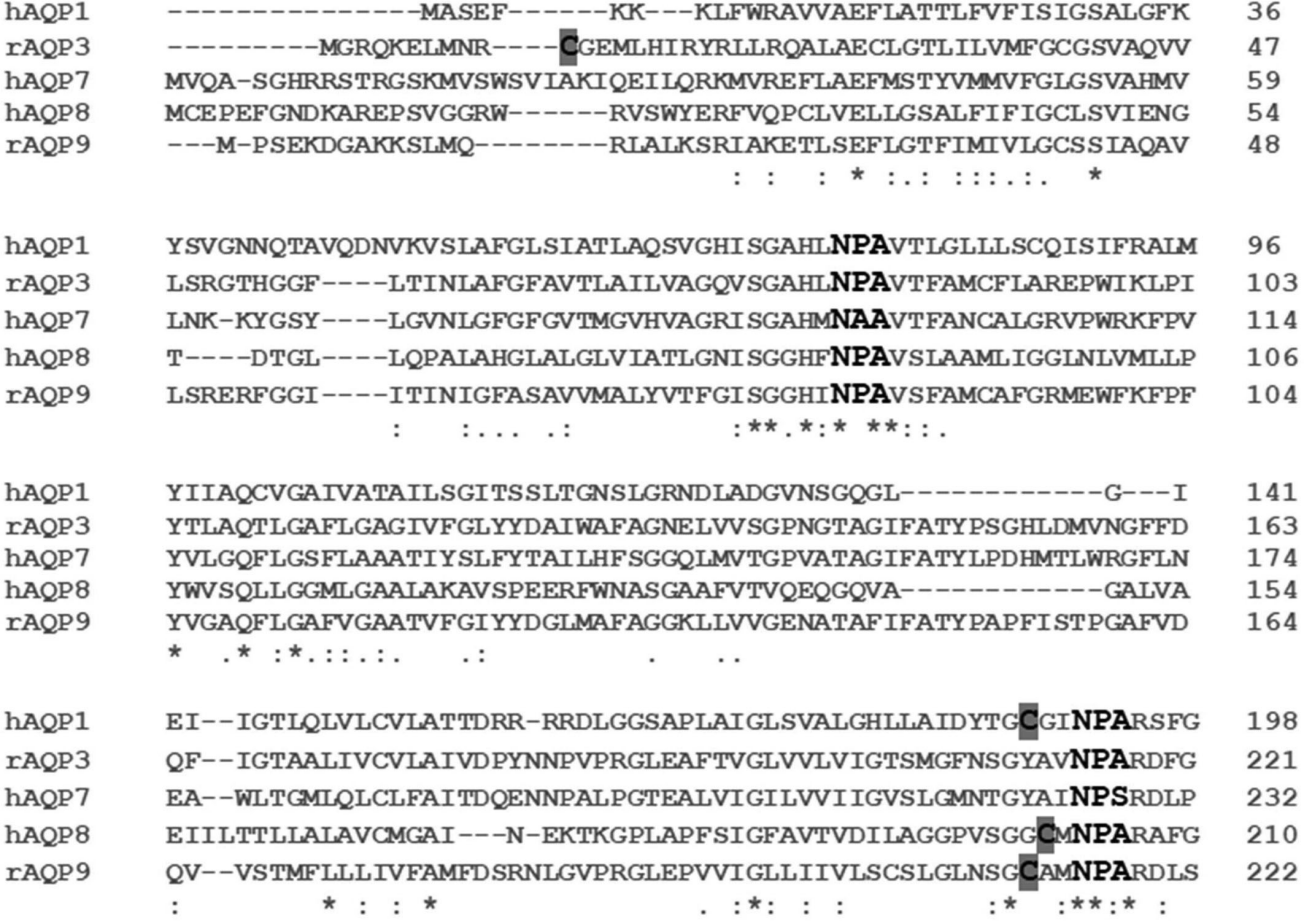
**Sequence alignment of hAQP1, rAQP3, hAQP7, hAQP8 and rAQP9.** The sequence alignment shows the two conserved NPA (asparagine-proline-alanine) motifs (bold), as well as the mercury-sensitive cysteine residues in AQP1 (Cys 189), AQP3 (Cys 11), AQP8 (Cys 202) and AQP9 (Cys 213) (highlighted in gray). Each Cys is located in the mouth of the aquapore, with the exception of the Cys in AQP3 (Cys 11), which is located on the intracellular side of the protein. AQP7 does not have a mercury-sensitive Cys residue, and is not inhibited by mercury.

Portions of an amino acid sequence alignment of the AQPs relevant to this study are shown in Fig. 2. The conservation of the two NPA motifs (signature of an AQP) and the site of the mercury-sensitive Cys (C) residues, which is near the second NPA motif and accessible from the extracellular side of the cell membrane, are highlighted in gray. Cys 189 in AQP1 is accessible from the extracellular surface and responsible for the mercury sensitivity. AQP8 and AQP9 possess an analogous Cys residue located in the throat of the aquapore (Cys 202 in AQP8 and Cys 213 in AQP9). Previous studies have shown that the mercury-sensitive Cys in AQP3 (Cys 11) is located on the intracellular surface of the protein (Kuwahara et al., 1997), and that AQP7 lacks a mercury-sensitive Cys, near the second NPA motif (Ishibashi et al., 1997b).

**Fig. 3.**
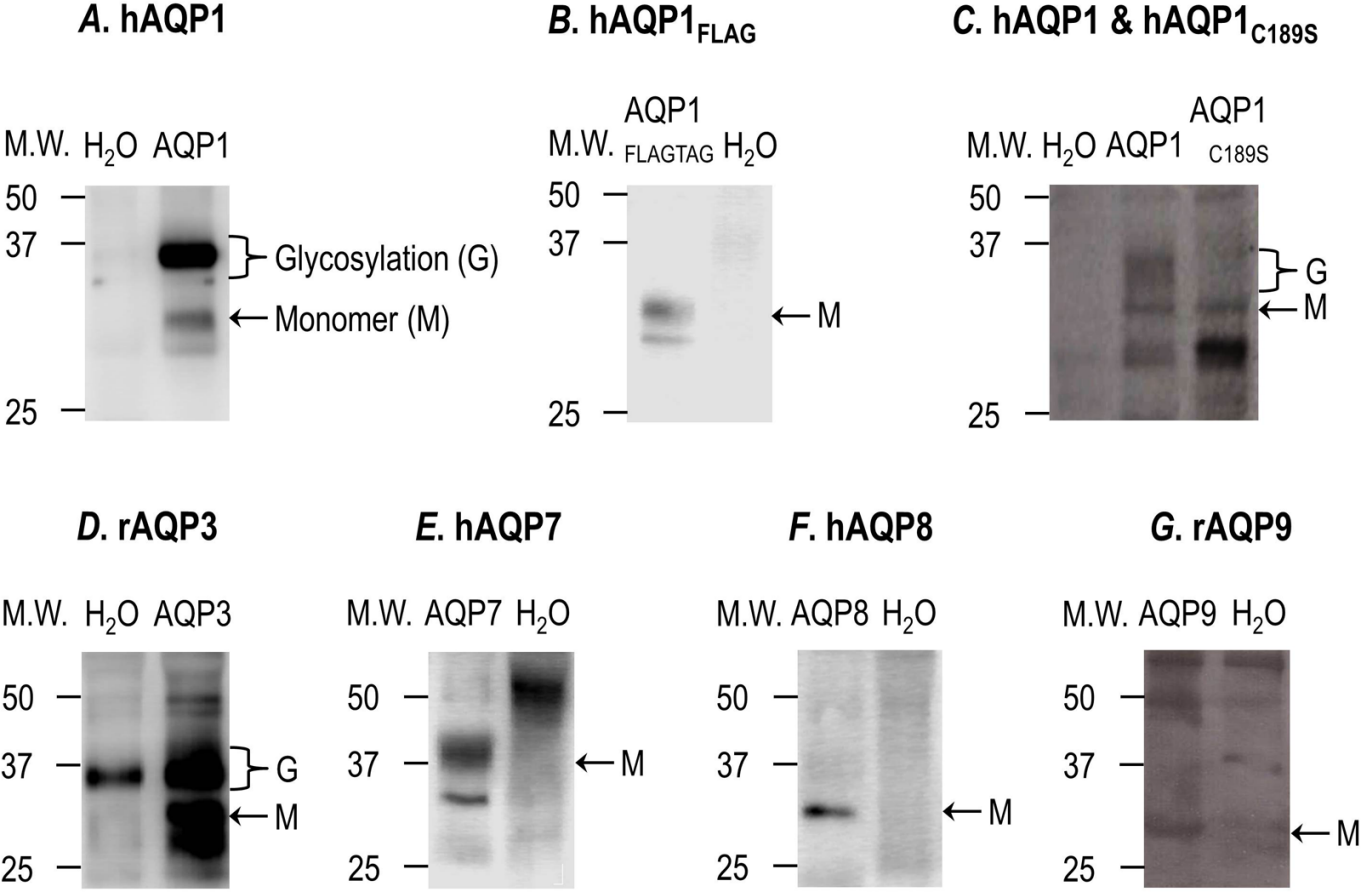
**Surface expression of hAQP1, hAQP1_FLAG_, hAQP1_C189S_, rAQP3, hAQP7, hAQP8 and rAQP9 versus H_2_O-injected control oocytes.** The surface expression of hAQP1, hAQP1_C189S_ mutant, rAQP3, hAQP7, hAQP8 and hAQP9 monomers (∼28 kDa) is shown by immunoreactive bands detected at a molecular weight (MW) between 25 and 37 kDa, using polyclonal antibodies (anti-AQP1, anti-AQP3, anti-AQP7, anti-AQP8 and anti-AQP9, respectively). The surface expression of hAQP1_FLAG_ monomer is shown by a band at a MW between 25 and 37 kDa, using a monoclonal antibody anti-FLAG. All the western blots show the absence of this immunoreactive bands in the H_2_O-injected control oocytes. Oocytes from 8–18 different frogs (i.e. batches of oocytes) were analyzed for surface expression, depending on the AQP construct used.

The western blots in Fig. 3 show that *Lithobates* oocytes injected with cRNA encoding for hAQP1 (Fig. 3A), hAQP1_FLAG_ (Fig. 3B), hAQP1_C189S_ mutant (Fig. 3C), rAQP3 (Fig. 3D), hAQP7 (Fig. 3E), hAQP8 (Fig. 3F) and rAQP9 (Fig. 3G) express the proteins at the cell surface. The immunoreactive AQPs bands are detected at a molecular weight (MW) between 25 and 37 kDa, which is indicative of monomeric AQPs (∼28 kDa) and glycosylated AQPs (∼37 kDa), with the exception of AQP7, which has a predicted MW of 37 kDa and does not have any consensus glycosylation sites (Ishibashi et al., 1997b). These results show that the AQP proteins were not only synthesized and posttranslationally modified but also inserted into the plasma membrane.

### Osmotic water permeability, *P*_f_

Having successfully demonstrated that *Lithobates* oocytes can translate and insert membrane proteins into the membrane, we next evaluated whether or not the expression of AQPs increases the *P*_f_ of oocytes.

**Fig. 4.**
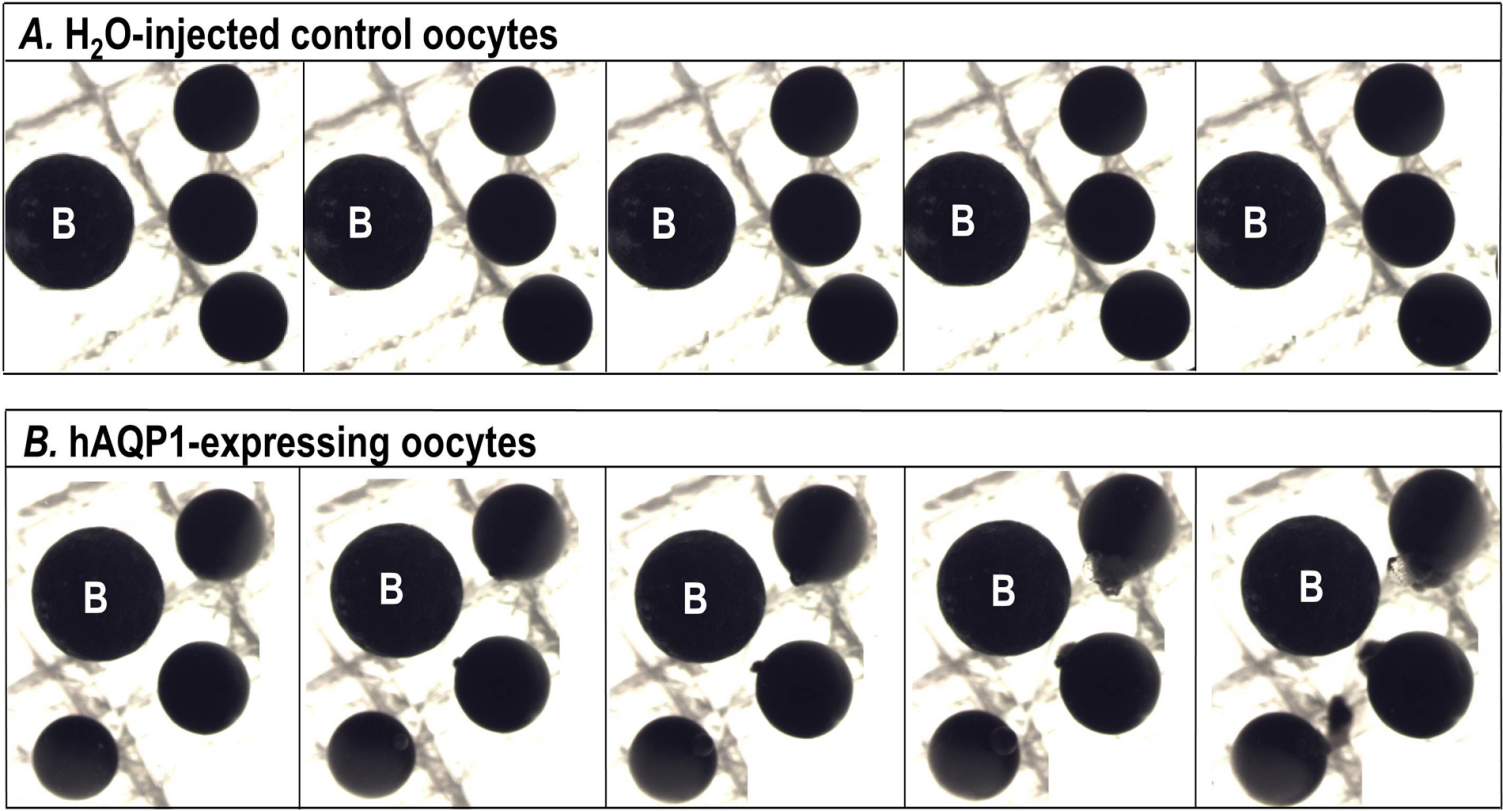
**Time course of cell swelling for hAQP1-expressing oocytes and H_2_O-injected controls.** (A) The pictures in the upper panel show three H_2_O-injected oocytes exposed to a hypotonic ND96 variant solution (∼70 mOsm) over the time course of 5 min, in which they do not show any significant cell swelling. (B) The pictures in the lower panel show three hAQP1-expressing oocytes exposed to the same hypotonic ND96 solution over the time course of 5 min, in which they show a significant cell swelling, as well as cell explosion. Values are means±s.e., with numbers of oocytes in parentheses.

Oocytes were injected with cRNA encoding for the AQPs or injected with water, as controls. Four days after injection, the oocytes were transferred from ND96 control solution (∼200 mOsm) to a hypotonic ND96 variant solution (∼70 mOsm) and the rate of oocyte swelling, from which we computed *P*_f_ (cm/s), was monitored using video microscopy. Fig. 4 shows photos of a typical time course of cell swelling for H_2_O-injected control oocytes (upper panel) and AQP1-expressing oocytes (lower panel) exposed to hypotonic ND96 variant solution over the course of 5 min. It is clear that, by the end of the time course, the membranes of oocytes expressing AQP1 started to break due to osmotic pressure. In contrast, the H_2_O-injected control oocytes did not swell.

**Fig. 5.**
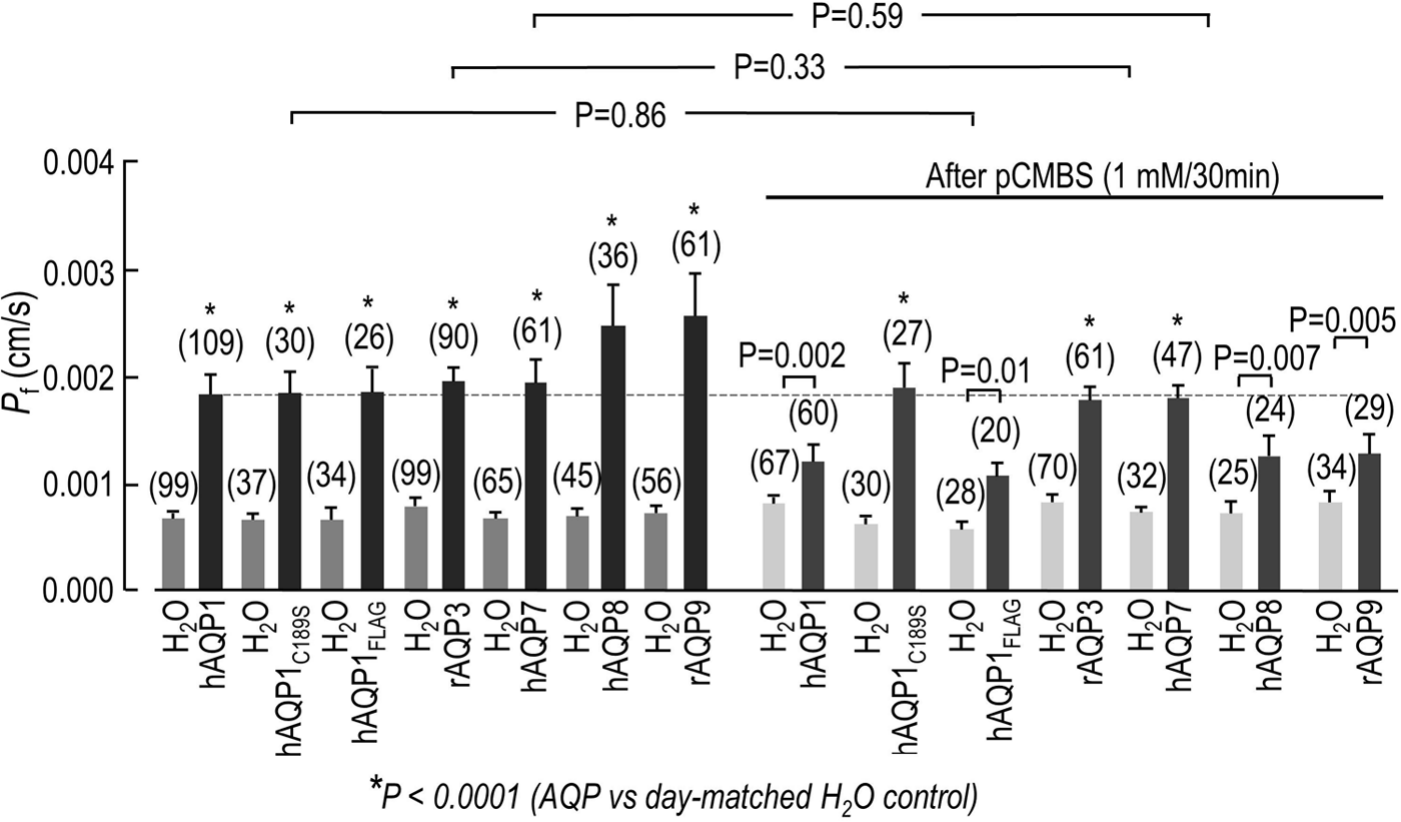
**Osmotic water permeability (*P*_f_) values of oocytes expressing hAQP1, hAQP1_C189S_, hAQP1_FLAG_, rAQP3, hAQP7, hAQP8 and rAQP9 versus their day-matched H_2_O-injected control oocytes, before and after the treatment with pCMBS (1 mM for 30 min).** The *P*_f_ values of AQP-expressing oocytes (black bars) are significantly greater than those of the *P*_f_ of day-matched H_2_O-injected controls (gray bars) (*P*<0.0001, *t*-test). Treatment with pCMBS significantly reduced the *P*_f_ of oocytes expressing AQP1 (*P*=0.002, *t*-test), AQP1_FLAG_ (*P*=0.01, *t*-test), AQP8 (*P*=0.007, *t*-test), and AQP9 (*P*=0.005, *t*-test) versus their day-matched H_2_O controls, but had no effect on oocytes expressing hAQP1_C189S_, rAQP3 and hAQP7 versus their day-matched H_2_O controls (*P*<0.0001, *t*-test). In addition, a comparison of hAQP1_C189S_ mutant, rAQP3 and hAQP7 before and after pCMBS treatment was not significant different based on a *t*-test (*P* values: 0.86 for hAQP1_C189S_, 0.33 for rAQP3 and 0.59 for hAQP7). Oocytes from 8–18 different frogs (i.e. batches of oocytes) were used for calculating the *P*_f_ of each AQP.

The rate of cell swelling, from which we calculate *P*_f_, was performed with each of the AQP-expressing oocytes versus its day-matched H_2_O-injected controls. Fig. 5 (left side) summarizes these experiments and shows that, oocytes expressing hAQP1, hAQP1_FLAG_, hAQP1_C189S_ mutant, rAQP3, hAQP7, hAQP8 and rAQP9 (black bars) displayed a mean *P*_f_ value that was significantly higher than that of day-matched H_2_O-injected controls (gray bars). To confirm that the increased *P*_f_ values were the result of AQP expression, the effect of the mercurial agent pCMBS on *P*_f_ was examined. The same oocytes were incubated with 1 mM pCMBS for 30 min, washed three times in ND96 solution, and then placed in the hypotonic ND96 variant solution (∼70 mOsm) and the rate of oocyte swelling, from which we computed *P*_f_, was again monitored using video microscopy. As shown in Fig. 5 (right side), pCMBS treatment reduced the *P*_f_ difference between hAQP1, hAQP1_FLAG_, hAQP8 and rAQP9 (dark-gray bars) and their day-matched H_2_O controls (light-gray bars), but had no effect on the *P*_f_ difference between hAQP_C189S_ mutant, rAQP3, and hAQP7 (dark-gray bars) and the day-matched H_2_O controls (light-gray bars). Together, these results indicate that all mammalian AQPs are functionally expressed in *Lithobates* oocytes, and that the enhanced water permeability is AQP-mediated, since the *P*_f_ of hAQP1, hAQP1_FLAG_, hAQP8 and rAQP9 can be inhibited by the mercurial agent pCMBS.

**Fig. 6.**
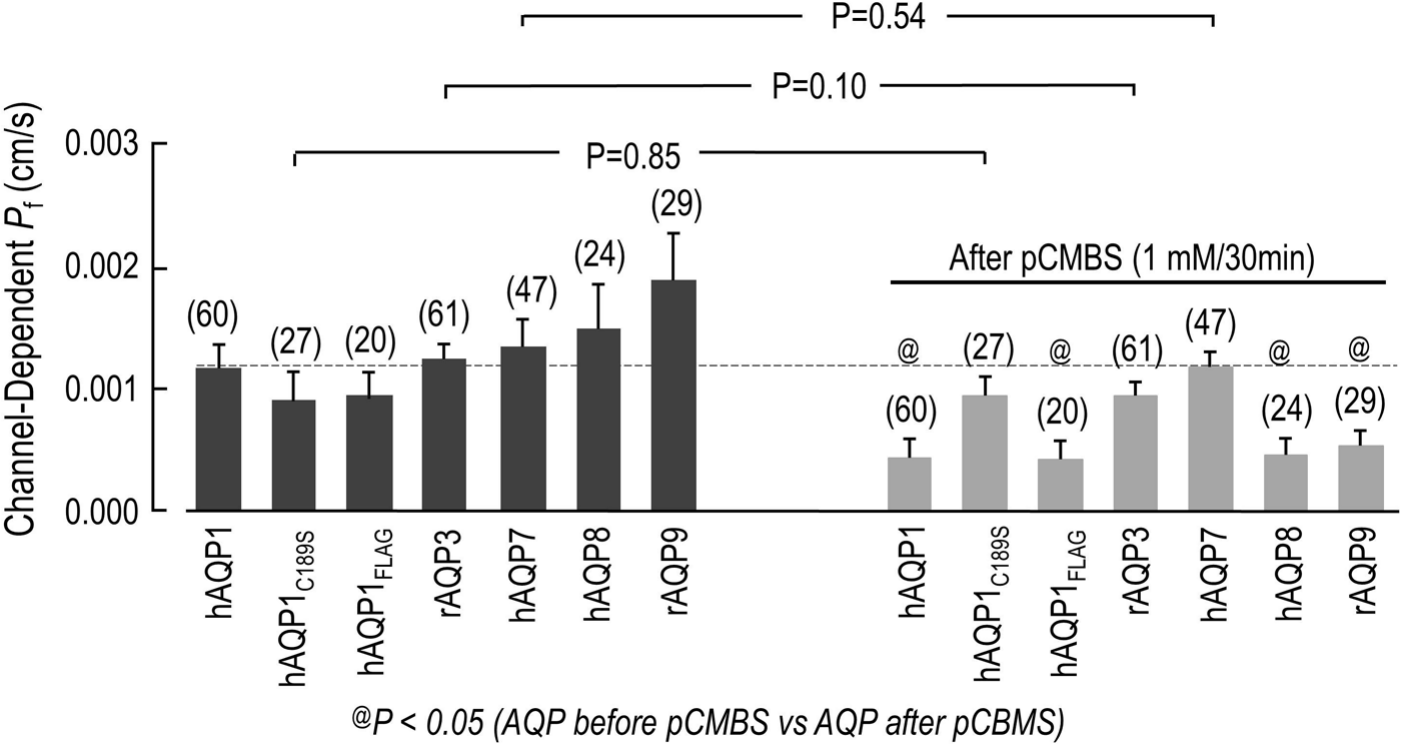
Channel-dependent osmotic water permeability (*P*_f_*) of oocytes expressing hAQP1, hAQP1_FLAG_ hAQP1_C189S_, rAQP3, hAQP7, hAQP8 and rAQP9 before and after treatment with pCMBS (1 mM for 30 min). Subtracting the *P*_f_ value for day-matched H_2_O-injected control oocytes from the *P*_f_ of each AQP-expressing oocyte, before and after pCMBS treatment yields the channel-dependent osmotic water permeability (*P*_f_*). Treatment with pCMBS reduces the *P*_f_* of hAQP1 (*P*=0.01, *t*-test), hAQP1_FLAG_ (*P*=0.03, *t*-test), hAQP8 (*P*=0.03, *t*-test) and rAQP9 (*P*=0.02, *t*-test), but has no effect on *P*_f_* of hAQP_C189S_ mutant (*P*=0.85, *t*-test), AQP3 (*P*=0.10, *t*-test), and AQP7 (*P*=0.54, *t*-test). A one-way ANOVA, followed by an SNK post hoc analysis to compare *P*_f_* before and after treatment with pCMBS was also performed. Overall *P* values: *P*=0.27 before pCBMS treatment and *P*=0.0001 after pCMBS treatment).

In Fig. 5, the portion of the *P*_f_ bar that we can ascribe to each AQP is the portion above the H_2_O-injected control background. Thus, we subtracted the mean *P*_f_ of each AQP-expressing oocyte from the mean *P*_f_ of the day-matched H_2_O control. This difference represents the channel-dependent *P*_f_ or *P_f_ of each AQP. Fig. 6 summarizes these differences and shows that pCBMS significantly reduces the *P_f_ of hAQP1, hAQP1_FLAG_, hAQP8 and rAQP9 by about half, but has no effect on the *P_f_ of hAQP1_C189S_ mutant (the mercury-insensitive AQP1), rAQP3 (that has the mercury-sensitive Cys located on the intracellular surface of the protein), and hAQP7 (that lacks a mercury-sensitive Cys).

## DISCUSSION

In this work, we set out to determine if oocytes isolated from *L. catesbeianus* can be utilized as an alternative to the *Xenopus* oocyte heterologous expression system. We found that the methodologies typically employed with *Xenopus* oocytes are not entirely compatible with *Lithobates* oocytes. Despite the blood parameters, of the two frogs, being quite similar, there are some unknown properties in the vitelline membrane and/or cellular membrane of the *Lithobates* oocytes. These differences in membrane properties cause the *Lithobates* oocytes to be extremely sensitive to Type IA collagenase, which is commonly used for the isolation of *Xenopus* oocytes (Musa-Aziz et al., 2010). The primary advantage of using the collagenase based method is that a large quantity of truly defolliculated oocytes can be obtained, relatively easily. However, collagenase treatment can affect the metabolic rate, and impact the robustness of the oocytes (Sive et al., 2000). In fact, under all Type IA collagenase concentrations and exposure times the *Lithobates* oocytes lysed and/or died. By evaluating the efficacy of three different types of collagenase, it was found that a brief 5 min treatment with the purified Type VII collagenase digestion was sufficient to effectively releasing individual oocytes, without removing the innermost layer of follicular cells and damaging the vitelline membrane, as evidenced by longer incubation times with all of the collagenases used, including Type VII. Therefore, following the brief 5 min Type VII collagenase treatment, it was necessary to mechanically or manually remove the follicular layer of cells surrounding the oocytes, so as to ensure that the vitelline membrane, which preserves the integrity and health of the oocyte (Musa-Aziz et al., 2010), remains intact.

While the technique to manually remove the follicular layer requires some level of training and practice (Sive et al., 2000), it routinely results in several hundred defolliculated and robust oocytes. By combining a brief 5 min treatment with the purified Type VII collagenase, followed by manual removal of the innermost layer of follicular cells, an effective method for isolating *Lithobates* oocytes was developed, which does not compromise the integrity of the cellular membrane or overall health of the oocyte.

Using methodologies for labeling and isolating proteins at the surface of the oocyte and evaluating AQP function, not entirely different from those utilized with *Xenopus* oocytes (Geyer et al., 2013a,b), we have also shown that *Lithobates* oocytes can express AQP proteins at the cellular surface and can significantly increase the *P*_f_ of the oocytes. Using the membrane-impermeable reagent EZ-link-sulfo-NHS-Biotin, it is possible to selectively modify and thus isolate proteins displaying lysine residues on the surface of the oocyte (Musa-Aziz et al., 2010). The experimental design takes advantage of the fact that all of our proteins have at least one extracellular lysine, which can react with the EZ-Link Sulfo-NHS-Biotin reagent. However, it is important to emphasize that the number of solvent accessible lysine residues in each AQP is not known, and suggests that the biotinylation efficiency may not be the same among all the AQPs. As shown in Fig. 3, all of the AQP proteins are expressed and detected at the surface of the oocyte membrane. Due to the fact that different polyclonal antibodies were utilized for the detection of each AQP, it was not possible to quantitate and normalize the expression of each AQP. To address this limitation, we worked with an AQP with a FLAG-tag inserted at the N-terminus (hAQP1_FLAG_), which can be detected with a monoclonal anti-FLAG antibody. As shown in Fig. 3B, hAQP1_FLAG_ was expressed at the membrane surface of *Lithobates* oocytes, and there were no non-specific immunoreactive bands observed. Furthermore, we confirmed that oocytes expressing hAQP1_FLAG_ were fully functional (significant increase in *P*_f_) and mercury sensitive, similar to oocytes expressing hAQP1 (Figs 5 and 6). It should also be noted that it appears as though the AQP1_C189S_ mutant had a higher apparent expression level than that of wild-type AQP1. This could be due to a variety of reasons such as higher translational rate of the cRNA, increased stability of the cRNA, increased stability of the translated protein, or resistance to proteolytic digestion, which will require a more analytical approach to answer and is beyond the scope of the present study.

The glycosylation of AQP1 and AQP3 was observed by the smeared band at a higher MW (∼37 kDa), and is consistent with what others have reported in the literature (Ishibashi et al., 1997a; Preston et al., 1992). AQP7 also displayed a band at ∼37 kDa; however, this protein has a predicted MW of ∼37 kDa, and does not contain a potential consensus glycosylation site (Ishibashi et al., 1997b). Thus, our interpretation of these results, coupled with the functional data, is that the immunoreactive band at ∼37 kDa represents monomeric AQP7, and the immunoreactive bands at a lower MW correspond to a proteolytic fragment of AQP7. Unexpectedly, no glycosylation was observed with AQP8 or AQP9, which is inconsistent with previous reports (Calamita et al., 2001; Viadiu et al., 2007). This anomaly could be attributed to the protein concentrations being below the limit of detection for observing the glycosylation product. Despite this discrepancy with AQP8 and AQP9 sites of glycosylation, functional expression (significant increase in *P*_f_) and mercury sensitivity were observed with both proteins (Figs 5 and 6).

When the AQP-expressing oocytes were placed in the hypotonic solution, significant changes in the oocyte volume were observed and reproducible *P*_f_ values were calculated (Fig. 5). Therefore, all AQPs used in this study display significant water permeability when expressed in *Lithobates* oocytes. To ascertain whether or not this significantly increased *P*_f_ was due to the presence of AQP channels being expressed at the surface of the oocyte, the mercurial agent pCMBS – which does not permeate cell membranes – was applied to the AQP-expressing oocytes and the day-matched H_2_O-injected controls. As shown in Fig. 5, the *P*_f_ difference between oocytes expressing AQP1, AQP1_FLAG_, AQP8, and AQP9 and their day-matched H_2_O controls were all significantly reduced, which is consistent with AQP1, AQP8 and AQP9 having a solvent-accessible mercury-sensitive Cys. The specificity of the pCMBS inhibitory effect was further confirmed with oocytes expressing AQP1_C189S_ mutant (the mercury-insensitive AQP1) and AQP7 (that lacks a mercury-sensitive Cys). Although C189S mutant and AQP7-expressing oocytes had a *P*_f_ equivalent to that of untreated oocytes, they were not inhibited by pCMBS (Figs 5 and 6). The results observed with *Lithobates* oocytes expressing AQP1_C189S_ mutant or AQP7 are therefore in agreement with previous studies using *Xenopus* oocytes. Preston et al. (1993) showed that mutating Cys 189 to a Ser had no effect on the AQP1-dependent osmotic water permeability (*P*_f_); however, this mutation does make AQP1 insensitive to mercury. Ishibashi et al. showed that AQP7 lacks a mercury-sensitive Cys in the throat of the aquapore, near the second NPA motif, and that it is resistant to mercury inhibition (Ishibashi et al., 1997b). On the other hand, and in contrast to previously published results, it was found that *Lithobates* oocytes expressing AQP3 are not mercury sensitive (Ishibashi et al., 1997a; Kuwahara et al., 1997).

Based on the location of the mercury-sensitive Cys in AQP3 on the intracellular surface of the protein and the properties of pCMBS, which does not permeate cell membranes (Vansteveninck et al., 1965), the mercury insensitivity is not completely unexpected. The first study investigating AQP3 mercury sensitivity was performed by (Kuwahara et al., 1997) and showed that AQP3 contains six Cys residues (at positions 11, 29, 40, 91, 174, and 267). This group showed that the mutation of each of the six Cys to a Ser revealed that only the cysteine at position 11 had resistance to mercury inhibition, which is localized on the intracellular side of AQP3. However, pCMBS is membrane impermeable (Vansteveninck et al., 1965). In fact, it has also been proposed that since mercury has to penetrate the cell membrane to reach its target, the inhibitory effect on AQP3 can be relatively slow (Zelenina et al., 2004). Therefore, inhibition would not be observed with AQP3, unless a significant amount of pCMBS crossed the membrane. It is also plausible that *Lithobates* oocyte membranes are less permeable to pCMBS, as compared to those of *Xenopus*.

## CONCLUSION

We found that *Lithobates* oocytes are a useful alternative to *Xenopus* oocytes, when investigating AQP proteins. *Lithobates* oocytes can express native and FLAG-tagged AQPs at the cell surface and increase the *P*_f_ of the oocytes. The augmentation of the *P*_f_ can be inhibited with pCMBS when the mercury-sensitive Cys in the aquapore is present and located on the extracellular side of the membrane. Future studies will further evaluate this heterologous expression system with other membrane proteins. Our findings are significant to any researcher that does not have unrestricted access to the *Xenopus* system.

## MATERIALS AND METHODS

### Expression in *Lithobates* oocytes

#### cRNA synthesis

Table 2 contains a list of the expression vectors, restriction enzymes and promoters used in this study. The plasmids encoding for hAQP1, hAQP1_FLAG_, hAQP1_C189S_ mutant, rAQP3, hAQP7, hAQP8 or rAQP9 were transformed into TOP10 competent cells. All of the plasmids were sequenced using the BigDye kit and the ABI Prism 3130XL Genetic Analyzer (Hitachi, Tokyo, Japan). Plasmid DNA was purified using either miniprep or midiprep kits (Qiagen). DNA concentration and purity were determined spectrophotometrically on a Nanodrop 2000c spectrophotometer (Thermo Fisher Scientific).

**Table 2.**
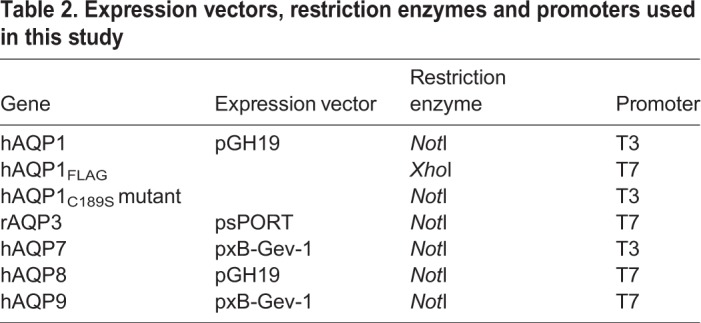
**Expression vectors, restriction enzymes and promoters used in this study**

Plasmids were linearized using the respective restriction enzyme (Table 2) and restriction-digested products were purified using the QIAquick PCR purification kit (Qiagen). The capped RNAs (cRNAs) were transcribed using either a T3 or T7 mMessage mMachine kit (Ambion, Austin, USA) depending on the promoter present on the linearized DNA (Table 2). The cRNA was then purified and concentrated using the RNeasy MinElute RNA Cleanup kit (Qiagen) and quantified by measuring the absorbance at 260 nm using a Nanodrop 2000c spectrophotometer (Thermo Fisher Scientific).

### Animals

All experimental procedures were approved by the Animal Care and Use Committee of the Institute of Biomedical Sciences of the University of Sao Paulo (protocol no. 83, page 35, book 3). All frogs were purchased from FROG Distribuidora De Rãs e Complementos Ltda-ME World (Sao Bernardo do Campo, Brazil) and housed in a frog facility under similar conditions of water temperature (22°C), room lighting (12:12-h light:dark cycle), and diet (Poli-Nutri, Sao Paulo, Brazil). All used animals were mature female frogs with weights ranging from 350 to 450 g.

### Surgery

Female *Lithobates* frogs were anesthetized by immersion in a solution of 3-aminobenzoic acid ethyl ester (Tricane 0.2%) (A5040, Sigma-Aldrich) in 5 mM Hepes, pH 7.5 until sufficiently anesthetized, as evidenced by toe-pinch, and then placed in an ice filled container, which functions as an additional anesthesia for amphibians. Ovary fragments containing oocytes were surgically removed by making a 1–1.5 cm incision through the skin and wall muscle in the abdomen, laterally to midline, and exposing the ovaries. Fragments of these ovaries were gently removed through the incision and placed into a 50 ml conical tube containing ND96 control solution (96 mM sodium chloride; see ‘Solutions’ section) for posterior oocyte isolation (see ‘Isolation of *Lithobates* oocytes’ section). In some cases, following the surgery, blood was collected by cardiac puncture for further analysis.

### Blood sample collection

Approximately 1.5 ml of blood was collected by cardiac puncture from the ventricle by using a 3 ml syringe (Becton Dickinson, Franklin Lakes, USA) and a 19-gauge needle (Becton Dickinson) and transferred to a heparinized tube. Collected blood was centrifuged for 10 min at 13,000×***g*** (Expresso Centrifuge, Thermo Fisher Scientific). After centrifugation, serum was pipetted into a new 1.5 ml microfuge tube and used immediately to measure Na^+^, Cl^−^, K^+^ concentrations (Electrolyte Analyzer AVL 9180, Roche, Mannheim, Germany), osmolarity (Vapro Vapor Pressure 5520, Wescor, South Logan, USA), pCO_2_ (partial pressure of carbon dioxide), HCO_3_^−^ and pH (Radiometer ABL5, Copenhagen, Denmark).

### Isolation of *Lithobates* oocytes

The ovary fragments – with oocytes wrapped by follicular cell layers containing blood vessels – were transferred to a 100 cm Petri dish containing ND96 control solution (see ‘Solutions’ section). Using sterile scissors, the fragments were further reduced into <5 mm^3^ pieces, while holding them with fine forceps. The small ovary pieces were washed three times (15 min/wash) in calcium-free solution (0-Ca^2+^ ND-96 solution; see ‘Solutions’ section) on the shaker, and then treated for 10 min with collagenase type VII (0.25 mg/ml) (Sigma-Aldrich) in 0-Ca^2+^ ND-96 solution on the shaker (Type 1A, Type II and Type V collagenase refered in the results section were also from Sigma-Aldrich.) The digestion process was monitored by routinely visualizing the oocytes under a dissecting microscope. The process was stopped by washing the oocytes with 0-Ca^2+^ ND-96 solution three times (15 min/wash), with ND96 control solution for 5 min, and then transferring the oocytes to ND96 control solution. Finally, using two watchmaker’s forceps, healthy-stage V-VI oocytes were mechanically defolliculated, sorted (based on size, stage and damage), and placed in a Petri dish containing OR3 culture medium (shown below in ‘Solutions’ section). After a final sorting step, the oocytes were transferred to a six-well culture plate containing OR3 medium, and stored at 18°C in sterile OR3 medium until used for injection.

### Microinjection of cRNAs

One day after isolation, oocytes were injected with 25 nl cRNA [25 ng (given as 25 nl of a 1 ng/nl cRNA solution)] encoding for hAQP1, hAQP1_FLAG_, hAQP1_C189S_ mutant, rAQP3, hAQP7, hAQP8 or rAQP9 or an equivalent volume of sterile water (called ‘H_2_O-injected control oocytes’). Sterile injection needles were made using a Model P-97 Flaming/Brown Micropipette Puller (Sutter Instrument Company, Novato, USA), as previously described (Musa-Aziz et al., 2010), and aseptically cut to have a diameter of approximately 2 μm. The needles were attached to a Nanoliter 2000 volume microinjector (World Precision Instruments, Sarasota, USA), filled with mineral oil and then filled with cRNA. Oocytes were injected with 25 nl cRNA or sterile water and stored at 18°C in OR3 medium. Expression and function were evaluated 3-5 days following injection.

### Solutions

The blood parameters indicated that no modifications of typical *Xenopus* solutions needed to be made. The ND96 control solution contained 96 mM NaCl, 2 mM KCl, 1 mM MgCl_2_, 1.8 mM CaCl_2_, and 5 mM HEPES (pH adjusted to 7.50 using NaOH or HCl). The modified zero calcium ND-96 solution (0 Ca_2_^+^ ND-96 solution) was made by replacing CaCl_2_ with NaCl (pH adjusted to 7.50 with NaOH or HCl). The OR3 culture medium contained 6.85 g/L of Leibovitz L-15 cell culture medium (L4386-1L, Sigma-Aldrich), supplemented with 10,000 U/ml penicillin G sodium, 10,000 mg/ml streptomycin sulfate (15140-122, Gibco, Gaithersburg, USA), and 5 mM HEPES (pH adjusted to 7.50 with NaOH). The osmolarity of all solutions was adjusted to between 195 and 200 mOsm (milliosmoles/L, the unit of osmotic concentration) with NaCl or water. For osmotic water permeability (*P*_f_) assays, a hypotonic ND96 solution variant (70 mOsm) was prepared by diluting the standard ND96 solution with water. For the inhibitory osmotic water permeability (*P*_f_) studies, the mercurial agent, p-chloromercuribenzene sulfonate (pCMBS) (C367750, Toronto 196 Research Chemicals, North York, Canada), was dissolved in ND96 control solution at a concentration of 1 mM (pH 7.50) immediately before the experiments (Geyer et al., 2013b).

### Surface expression measurements

#### Biotinylation

The surface protein expression was determined using the EZ-Link Sulfo-NHS-Biotinylation kit (89881, Thermo Fisher Scientific) according to the manufacturer's recommendations with some modifications (Geyer et al., 2013a). Prior to surface tagging, the phosphate-buffered saline (PBS, 1890535, Thermo Fisher Scientific) and Tris-buffered saline (TBS, 28376, Thermo Fisher Scientific) were diluted to reduce the osmolality of the solutions from 300 mOsm to 200 mOsm, thus matching that of the other oocyte solutions, and ensuring that the oocytes would not be exposed to a hyper-osmotic condition. Briefly, groups of ∼20 oocytes – expressing hAQP1, hAQP1_FLAG_, hAQP_C189S_ mutant, rAQP3, hAQP7, hAQP8, rAQP9 or H_2_O-injected controls – were incubated in 5 ml PBS +0.24 mg/ml of the membrane impermeable EZ-link-sulfo-NHS-Biotin (Thermo Fisher Scientific) for 1 h. The reaction was stopped by adding 250 μl of the supplied Quenching solution. The oocytes were washed in TBS for 5 min. The oocytes were then transferred to 200 μl lysis buffer (TBS, 1% TX-100, and cOmplete Mini EDTA-free protease inhibitor tablet; 04693124001, Roche, Indianapolis, USA), and manually lysed by pipetting the cells up and down using a P200 pipette tip. The lysate was centrifuged at 3000×***g*** for 10 min and the supernatant was removed. Twenty microliters of supernatant was mixed 1:1 with 2× sample buffer. This sample contains protein from both extracellular and intracellular expression, thus representing total expression. The remainder of the supernatant (∼180 μl) was transferred to a Spin X column (8163, Corning, Pittston, USA) containing 180 μl NeutrAvidin Gel (Thermo Fisher Scientific). The samples were mixed on a rocker platform for 1 h at room temperature and washed three times with lysis buffer. Finally, 180 μl of the 1× sample buffer containing 50 mM DTT was applied to the NeutrAvidin Gel-biotin labeled protein mixture, and continuously mixed on a rocker platform for 1 h at room temperature. Samples were collected by centrifugation at 1000×***g*** for 1 min. This final eluted sample represents the surface fraction.

#### Western blot analysis

The total and surface biotinylated samples from oocytes AQP injected with cRNA encoding for AQPs or H_2_O-injected controls, were separated by SDS-PAGE on 12% Tris-glycine gels. The samples were transferred to PVDF membranes and incubated in TBST with 5% of milk for 1 h at room temperature. The membranes were probed overnight at 4°C with polyclonal anti-hAQP1 (AQP11-A, Alpha Diagnostics, San Antonio, USA), anti-rAQP3 (AQP31-A, Alpha Diagnostics), hAQP7 (AQP71-A, Alpha Diagnostics), anti-hAQP8 (AQP81-A, Alpha Diagnostics), anti-hAQP9 (AQP91-A, Alpha Diagnostics) or monoclonal anti-FLAG (F3165-2MG, Sigma Aldrich) antibodies. The protein expression was detected using ECL plus Western Blotting Detection Reagents (32132, Thermo Fisher Scientific) and the signals were detected on an Amersham Imager 600 (GE Healthcare).

### Physiological measurements

#### Measurement of osmotic water permeability of oocytes, *P_f_*

We determined *P*_f_ using a volumetric assay (Preston et al., 1993; Virkki et al., 2002). Briefly, we placed a group of up to six oocytes into a Petri dish containing the aforementioned 70 mOsm ND96 variant solution and a metallic sphere, set next to the oocytes served as a size reference. Osmotic swelling was monitored with a Nikon stereoscopic microscope (SMZ 745T) equipped with a digital camera (Optix Cam, Roanake, USA) connected to a computer to monitor the projection area of each oocyte over time. Video images were acquired every 10 s for 5 min. The change in the projected area over time was used to calculate the osmotic water permeability (*P*_f_, cm/s) of the oocytes (Preston et al., 1993). For the inhibition experiments, the oocytes were preincubated in ND96 plus 1.0 mM of the mercurial agent pCMBS (195 mOsm, pH 7.50), which is known to reduce the *P*_f_ of AQP1 (Preston et al., 1993), for 30 min and, then, exposed to the hypotonic ND96 variant solution (70 mOsm, pH 7.50).

In order to compute *P*_f_, we assumed the oocyte to be a sphere with a true surface area (S) eightfold greater than that of the idealized sphere (Chandy et al., 1997), according to the equation:
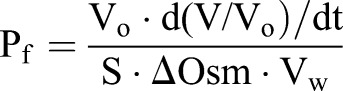
where V_o_ is the initial oocyte volume, d(V/V_o_)/dt is the maximal fractional rate of volume increase, ΔOsm is the osmotic gradient across the plasma membrane (195 mOsm_(inside)_–70 mOsm_(out)_=125 mOsm), and V*_w_* is the molar volume of water (18 cm^3^/mol) (Preston et al., 1992).

### Statistics

All data are presented as mean±s.e., with numbers of oocytes in parentheses. When comparing the difference between two means, Student's *t*-tests (two tailed) were performed. When comparing the difference among more than two means, one-way ANOVA followed by a Student–Newman–Keuls (SNK) post hoc analysis was performed using KaleidaGraph (version 4; Synergy Software, Reading, USA). *P*<0.05 was considered significant.
